# Affective Immunology: The Crosstalk Between Microglia and Astrocytes Plays Key Role?

**DOI:** 10.3389/fimmu.2020.01818

**Published:** 2020-08-20

**Authors:** Linglin Yang, Yunxiang Zhou, Honglei Jia, Yadong Qi, Sheng Tu, Anwen Shao

**Affiliations:** ^1^Department of Psychiatry, The Second Affiliated Hospital, Zhejiang University School of Medicine, Hangzhou, China; ^2^Department of Surgical Oncology, The Second Affiliated Hospital, Zhejiang University School of Medicine, Hangzhou, China; ^3^Department of Student Affairs, Zhejiang University School of Medicine, Hangzhou, China; ^4^Department of Gastroenterology, Sir Run Run Shaw Hospital, Zhejiang University School of Medicine, Hangzhou, China; ^5^State Key Laboratory for Diagnosis and Treatment of Infectious Diseases, Collaborative Innovation Center for Diagnosis and Treatment of Infectious Diseases, The First Affiliated Hospital, College of Medicine, Zhejiang University, Hangzhou, China; ^6^Department of Neurosurgery, The Second Affiliated Hospital, Zhejiang University School of Medicine, Hangzhou, China

**Keywords:** astrocyte-microglia crosstalk, neuroinflammation, mood disorders, depression, bipolar disorder

## Abstract

Emerging evidence demonstrates the critical role of the immune response in the mechanisms relating to mood disorders, such as major depression (MDD) and bipolar disorder (BD). This has cast a spotlight on a specialized branch committed to the research of dynamics of the fine interaction between emotion (or affection) and immune response, which has been termed as “affective immunology.” Inflammatory cytokines and gut microbiota are actively involved in affective immunology. Furthermore, abnormalities of the astrocytes and microglia have been observed in mood disorders from both postmortem and molecular imaging studies; however, the underlying mechanisms remain elusive. Notably, the crosstalk between astrocyte and microglia acts as a mutual and pivotal intermediary factor modulating the immune response posed by inflammatory cytokines and gut microbiota. In this study, we propose the “altered astrocyte-microglia crosstalk (AAMC)” hypothesis which suggests that the astrocyte-microglia crosstalk regulates emotional alteration through mediating immune response, and thus, contributing to the development of mood disorders.

## Introduction

Almost a century ago, Julius Wagner-Jauregg first reported the impact of immunological disturbance on psychological function. This finding stirred up researchers' enthusiasm about bidirectional communication between immunological dysfunction and mental disorders. To date, understanding the etiology of mental disorders, such as schizophrenia, autism spectrum disorder, anxiety disorder, as well as mood disorders, is one of the most actively explored topics in immunology (1, 2).

Mood disorders, including major depressive disorder (MDD) and bipolar disorder (BD), are a group of complex debilitating psychiatric illnesses identified by symptoms rather than biological markers. Both of these disorders remain serious health concerns worldwide, owing to their high prevalence, risk for recurrence, and suicide. Further, the mainstream pharmacological treatments—antidepressants and mood stabilizers—are unsatisfactory in treating such patients due to their delayed onset of action, limited efficacy, and vast array of adverse side effects (3, 4). The important reason behind this dilemma is frustratingly limited understanding of the pathological mechanism underlying mood disorders, including affective immunology (5, 6).

To understand the dynamics of the fine interaction between the emotion (or affection) and immune response, a specialized branch called “affective immunology” was recently introduced to distinguish it from “psychoneuroimmunology” which broadly studies the relationship between psychological processes, neuroendocrine activities, and immune systems (7). The high plasticity of the immune system significantly raises the exciting possibility of translational research. In the wake of rapidly accumulating evidence implying the critical role of affective immunity in the cellular and molecular mechanisms underlying the mood disorders, great efforts are ongoing to develop more immunomodulators targeting immune cells (especially microglia) (8, 9), inflammatory cytokines (10, 11), as well as gut microbiota (12, 13).

Microglia are widely known as innate sentinel immune cells that reside in the central nervous system (CNS). These cells respond dynamically to changes in the physical environment and are proven to be key players in affective immunology. Additionally, another common glia, astrocytes, also participate in neuroinflammation by re-sculpting blood-brain barrier (BBB) and releasing inflammatory cytokines. Interestingly, a unique bond between microglia and astrocytes exists, namely the astrocyte-microglia crosstalk, coordinate their functions in neuroinflammatory response (14). Collectively, the astrocyte-microglia crosstalk likely exerts an influence on emotion and affection by regulating the neuroinflammatory response. Hence, we speculate that the altered astrocyte-microglia crosstalk (AAMC) is a primary determinant of mood disorders, thereby a more specific and direct therapeutic target.

In this study, we integrate available data from both preclinical and translational studies regarding affective immunology and highlight the core role of the astrocyte-microglia crosstalk. Furthermore, we intend to discuss the dysregulated crosstalk between astrocytes and microglia and hope to shed some light on potential therapeutic opportunities for treating mood disorders.

## Affective Immunology

### The Interaction Between Emotion and Immune System

Researchers have revealed that emotion and immune system mirror each other. However, the effect and causality between the two are debatable.

Mounting evidence has shown the beneficial impact of positive emotion (e.g., humor, happiness, and hope) on the immune system. For instance, people with positive emotions showed lower susceptibility to infection (15, 16) and greater immune response (17, 18). This role of “immune enhancer” is mediated by higher levels of antibodies (18) and T cells (19–21), and increased activity of natural killer (NK) cells (19, 22), as well as reduced inflammatory markers including interleukin-6 (IL-6) and C-reactive protein (CRP) (22, 23). In contrast, negative emotions (e.g., sadness, nervousness, worry, loneliness, and fear) and psychological stress are associated with poorer immunological function with lower NK cell cytotoxicity (8), fewer T cells (24), and increased inflammatory markers (25). Importantly, these immune alterations can persist up to 2 h following brief emotional turbulence like stress exposure (26), and it might be stored as immunological memory, thus shedding light on the development of emotional interventions (27).

Contrastingly, some researchers suggest that a dysfunctional immune system can induce emotional changes. For instance, immunotherapy using interferon-alpha (IFN-α) and vaccination can lead to negative emotion along with increased levels of inflammatory cytokines (10, 28, 29). Particularly, these cytokines are postulated to regulate BBB permeability and activity of the hypothalamic-pituitary-adrenal (HPA) axis (30, 31). This thus influences various neuronal events related to emotions including glia-neuron communication, neurotransmission, and synaptic pruning (32, 33). This finding leads to an interesting hypothesis that emotions can act as an “infection defense” to various environmental pathogens (34).

Taken together, bidirectional communication between emotions and the immune system exists, suggesting a striking role of the immune response in the development of mood disorders.

### The Compelling Role of Inflammation in Mood Disorders

Aberrant inflammatory processes exert an influence on the progression of mood disorders and also mediate the treatment response. High levels of peripheral inflammatory cytokines and chemokines, including IL-1β, IL-6, IFN-γ, tumor necrosis factor-alpha (TNF-α), and CRP, have been reported in patients with depression (35). In addition, the levels of these cytokines are reduced following an effective antidepressant therapy (36). Conversely, anti-inflammatory treatment results in improvement of depressive symptoms (37). Furthermore, immune-related genes encoding these cytokines have recently been found to be associated with depression (38), thus strongly supporting postulation that inflammatory cytokines are the active regulator of depression. Therefore, inflammatory cytokines are considered as “biomarkers” for diagnosis, susceptibility, and treatment responsiveness for MDD (36). With regards to BD, inflammatory cytokines have been found to be upregulated in both the depressive and manic episodes and return to normal levels in the euthymic state (9, 39). In addition to circulating cytokines, transmembrane TNF-α has also been shown to significantly increase in Brodmann area (BA) 46 for MDD and BA24 for BD (40).

Other than cytokines, the emerging evidence shows altered composition of gut microbiota in both MDD and BD (13, 41, 42). Specifically, *Alistipes* and *Klebsiella* are increased in MDD patients, while bacteria belonging to the *Lachnospiraceae* family are decreased (12, 43). Likewise, an abundance of *Lachnospira* was reported in the gut of BD patients (44). The altered gut microbiome can not only influence peripheral immune response (45) but can also regulate the neuroinflammation via the vagus nerve and microbial metabolites such as short-chain fatty acids (SCFA), secondary bile acids, serotonin, tryptophan metabolites (46), and neurotransmitter production (e.g., gamma-aminobutyric acid, noradrenaline, dopamine, and acetylcholine) (47, 48). However, since there is large variability in the composition and diversity of gut microbiota between individuals and emotional states, it remains difficult to identify an optimal microbiome profile. A major challenge lies in translating these observations into interventions that could be used to treat mood disorders.

**Figure 1:**
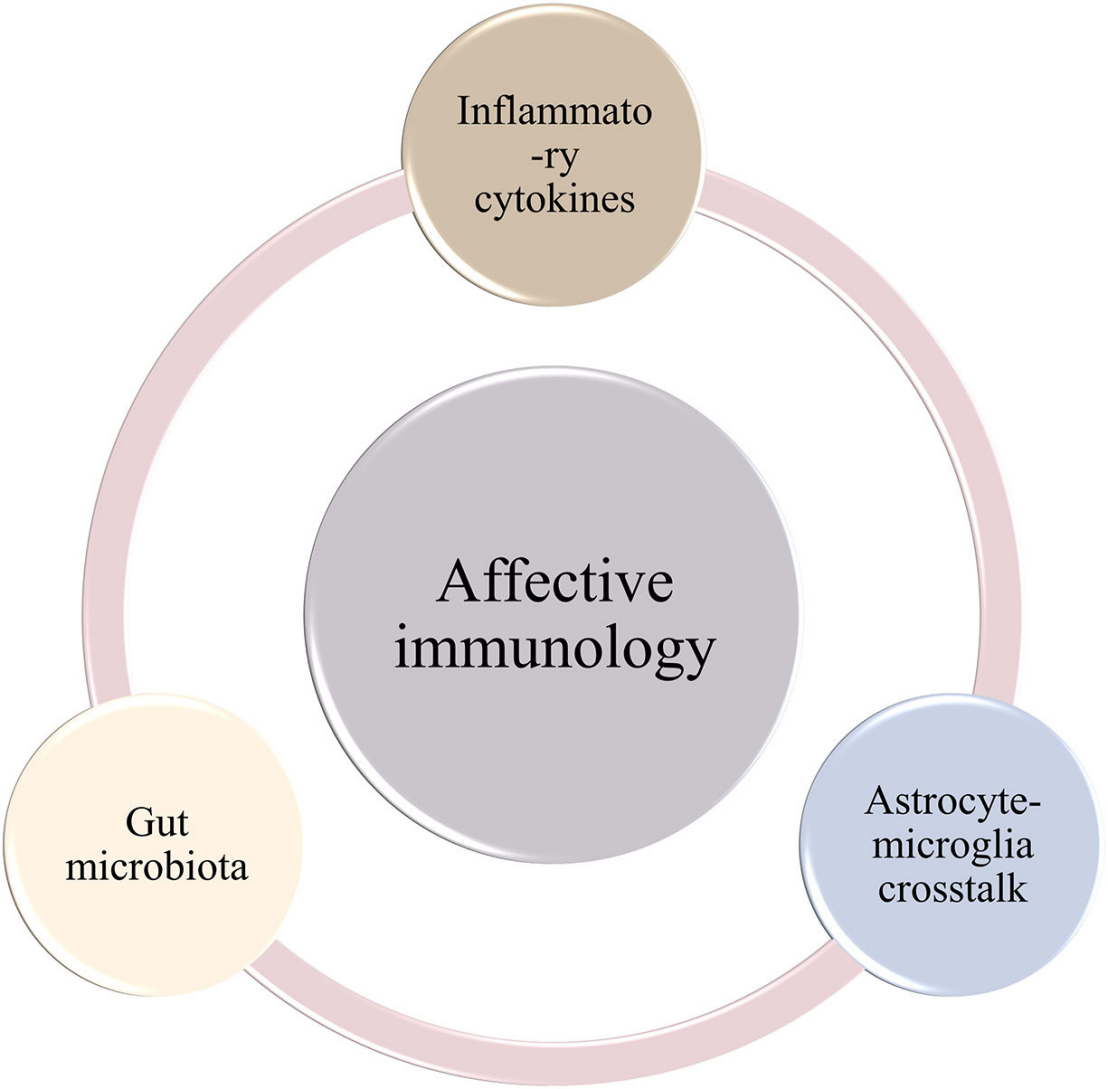
Schematic illustration of three cornerstones of affective immunology.

Taken together, the inflammatory cytokines and gut microbiota play compelling roles in the etiology of mood disorders (Figure 1). Particularly, both have profound impacts on the neuroinflammatory response by boosting the activity of microglia and astrocytes (46, 49, 50). In the next section, we present pathological alterations of both microglia and astrocytes in depression and BD, respectively.

## Dysfunction of Microglia and Astrocytes in Mood Disorders

Dysfunctional astrocytes and microglia are inextricably intertwined in mood disorders. To date, a growing body of evidence from human autopsy and serum/CSF/imaging biomarkers indicates their abnormalities underlying mood disorders (Table 1). Additionally, distinct gene profiling patterns of these cells have been reported. For instance, the *CD206* gene expression pattern of microglia varies between depressive and manic states of BD, suggesting the genetic evidence of microglial dysfunction in BD and the potential of microglial *CD206* as a state marker (73).

**Table 1:** Astrocytic and microglial markers in mood disorders.

**Molecular markers**	**Cell type**	**Human cohort studied**	**Sample studied**	**Main findings**	**References**
GFAP	Astrocyte	MDD	Human postmortem tissue	Decreased in amygdala, cerebellum, hippocampus, PFC (including BA10), cingulate cortex, thalamus and caudate	(51–56)
				Increased in basal ganglia	(57)
				No significant difference in ACC, PFC, entorhinal cortex, hippocampus and corpus callosum	(58–61)
		BD	Human postmortem tissue	Decreased in PFC (including BA10/11/47) and BA24	(53, 62, 63)
				Increased in PFC (including BA9)	(64–66)
				No significant difference in amygdala, cerebellum, ACC, PFC (including BA 9/10/46), BA40, basal ganglia, entorhinal cortex and corpus callosum	(51, 52, 57–61, 67, 68)
S100β	Astrocyte	MDD	Human postmortem tissue	No significant difference in amygdala	(69)
				Decreased in hippocampus	(61)
		BD	Human postmortem tissue	Decreased in hippocampus and BA 9	(61, 68)
				No significant difference in amygdala	(69)
				Increased in BA40	(61)
		BD (manic state)	Serum	Increased in serum	(70)
ALDH1L1	Astrocyte	MDD	Human postmortem tissue	Increased in basal ganglia	(57)
		BD	Human postmortem tissue	No significant difference in basal ganglia	(57)
HLA-D	Microglia	MDD	Human postmortem tissue	No significant difference in PFC, ACC, mediodorsal thalamus, hippocampus and amygdala	(69, 71)
		BD	Human postmortem tissue	No significant difference in PFC, ACC, mediodorsal thalamus, hippocampus and amygdala	(69, 71)
		Unipolar and bipolar depression	Human postmortem tissue	Decreased in dorsal raphe nucleus (non-suicidal subgroup)	(72)
CD206	Microglia	BD	Peripheral blood	Downregulated in the manic state	(73)
MCP-1/CCL-2	Microglia and astrocytes	BD (euthymic state)	Serum, CSF	Increased in both serum and CSF	(74)
YKL-40/CHI3L1	Microglia	BD (euthymic state)	Serum, CSF	Increased in both serum and CSF	(74)
sCD14	Microglia	BD (euthymic state)	Serum, CSF	Increased in serum while no significant difference in CSF	(74)
CD11B	Microglia and astrocytes	BD	Human postmortem tissue	Decreased in ACC	(75)
				No significant difference in frontal cortex	(40)
				Increased in PFC	(65)
IBA-1	Microglia	BD	Human postmortem tissue	No significant difference in BA9	(64)
TSPO	Microglia	MDD (mild to moderate depression)	[11C] PBR28 PET	No significant difference	(76)
		MDD (severe depression)	[^18^F] FEPPA PET	Increased in PFC, ACC and insula	(77, 78)
		MDD (late-life)	[11C] PK11195 PET	Increased in ACC and hippocampus	(79)
		BD (euthymic state)	[11C] PK11195 PET	Increased in hippocampus	(80)
Quinolinic acid	Microglia	Unipolar and bipolar depression	Human postmortem tissue	Increased in cingulate cortex	(32)

*MCP, monocyte chemoattractant protein; CCL, C-C motif chemokine ligand; MDD, major depression disorder; BD, bipolar disorder; PFC, prefrontal cortex; ACC, anterior cingulate cortex; TSPO, translocator protein; GFAP, Glial fibrillary acidic protein; CSF, cerebrospinal fluid; BA, Brodmann area; HLA-D, Human leukocyte antigen D; CHI3L1, chitinase-3-like protein 1; IBA-1, ionized calcium-binding adapter molecule-1; PET, positron emission tomography*.

### Dysfunction of Microglia and Astrocytes in Depression

Postmortem brain tissue of suicide victims has provided evidence suggesting enhanced microglial activation in the depressive episode (32, 71, 81). Steiner et al. observed greater human leukocyte antigen, D related (HLA-DR) staining in the dorsolateral prefrontal cortex (PFC) and anterior cingulate cortex (ACC) (71). Subsequently, an increased density of microglial quinolinic acid within ACC was reported in both unipolar and bipolar depression (32). Strikingly, it was found that primed microglia, rather than resting phenotype, were increased in ACC (81). These findings are consistent with the findings of recent molecular imaging studies using translocator protein (TSPO) as a marker of microglial activation (77, 78). Elevated TSPO density was found in PFC, ACC, and insula of patients experiencing major depressive episode (77, 79), especially in those with a long duration of untreated MDD (78). However, an earlier positron emission tomography (PET) imaging studies showed no significant difference in TSPO density between depressive patients and matched controls (76). The discrepancies may be related to the relatively small sample size and high heterogeneity of severity, onset age, and antidepressants used. Collectively, enhanced microglial activation in specific brain regions is a core constituent of depression pathology. Accordingly, inhibition of microglial activation by minocycline administration can lead to an improvement in depressive symptoms in various animal and human studies (31, 82–85). Similarly, blocking the adenosine triphosphate (ATP)-gated P2X7 ion channel of microglia was shown to be a potential, new, and effective antidepressant therapy (86).

Morphological and functional abnormalities of astrocytes have also been seen in patients with depressive episode. A histological study using Golgi-staining found hypertrophic astrocytes with more intricate processes and longer projections within ACC of depressed suicide cases (87), suggesting local low-grade inflammation with reactive astrocytosis. These findings were further confirmed by the observation of weakened BBB with reduced astrocytic endfeet (88) and gap junction proteins (89, 90), which facilitates the recruitment of immune cell and diffusion of pro-inflammatory cytokines (30). In parallel, mounting evidence of astrocyte-specific biomarkers demonstrates the dysfunction of astrocytes in depressive episode. Glial fibrillary acidic protein (GFAP), involved in astrocytic structure and movement, is thought to be upregulated during neuroinflammation (91). However, decreased density of GFAP-positive astrocytes was consistently found in depression-related brain regions, such as PFC, cingulate cortex (55, 58), hippocampus (54), amygdala (51), thalamus, and caudate nuclei (56). Although of less astrocyte-specificity, other markers such as calcium-binding protein S100β (92) and the water channel aquaporin-4 (AQP-4) (93) provided supporting evidence of astrocytic damage (especially neuroprotective phenotype) during dysregulated neuroinflammatory response induced by depressive episode (61, 88). Importantly, possibly due to epigenetic mechanism, maternal depression can result in a profound reduction of astrocyte density in the offspring, as shown in an animal model (94). In summary, the pathological alterations in astrocytes represent a prominent characteristic of depression, which can be reversed using effective antidepressant therapy. Fluoxetine (63, 95, 96), mirtazapine (97), ketamine (98, 99), as well as repetitive high-frequency transcranial magnetic stimulation (TMS) (100) have been shown to have a beneficial impact on astrocytes, paralleled by improvement of depressive symptoms. Additional support for the critical role of astrocytes in depression is derived from recent studies suggesting the therapeutic option for depression via the regulating the activity of astrocytes (101, 102).

### Dysfunction of Microglia and Astrocytes in Bipolar Disorder

Although the dysfunction of microglia and astrocytes has also been implicated in the development of BD (103, 104), the picture appears to be more complicated compared to MDD. Human postmortem studies in BD have not yielded consistent results. The majority of them showed an unchanged density of astrocytes and microglia in the frontal cortex (40, 64, 105, 106), ACC (60), amygdala (51, 67, 69), hippocampus (107), entorhinal cortex (59, 67), basal ganglia (57), dorsal raphe nucleus (72), and cerebellum (52). On the contrary, several studies showed positive results. For instance, the level of GFAP has been reported to be increased in BA9 (66) and decreased in BA10 (53), BA24 (63), BA11, and 47 (62). The level of S100β has been reported to be increased in BA40 and decreased in BA9 (68). The expression of CD11b protein, a marker of astrocytic and microglial activation, has been reported to be upregulated in PFC (65) and downregulated in ACC (75).

Obviously, the heterogeneity in terms of brain regions studied (62, 68) and methodology used (40, 66, 107) contributes to the discrepancy in these findings. Additionally, the mixed perimortem states are conceivably confounding factors that cannot be neglected. Some brain tissues were from depressive suicide cases, while most were from patients that died due to physical disease including pneumonia, pulmonary embolism, myocardial infarction, and cerebral hemorrhage which might affect acid-base balance and neuroinflammatory response (64, 72, 107). Also, substance abuse is common in BD and it can influence microglial activity (75). For each subject, the diagnosis of BD was based on the retrospective review of medical records and extensive telephone interviews with relatives, but their comorbidity and phenotype (depressive episode, manic episode or remission state) remain unclear (108). Due to the complexity of BD, studies regarding its diverse phenotypes are requisite to identify trait- or state-related alterations of astrocytes and microglia (5). Similar to unipolar depression, bipolar depression has been found to be related to reduced S100β positive astrocytes in the bilateral hippocampus (CA1 subregion) (61). Nevertheless, no significant difference in GFPA positive astrocyte and HLA-DR positive microglia were found in bipolar depression, which might be ascribed to the relatively small sample size (61, 71). With regards to manic episode, higher levels of peripheral S100β have been observed, implying astrocytic activation (70). Likewise, astrocytic and microglial activation are involved in euthymic patients (74, 80). Jakobsson et al. found increased cerebrospinal fluid (CSF) and serum levels of MCP-1/CCL2 and YKL-40/chitinase-3-like protein 1 (CHI3L1) in patients with mood-stabilized BD (74). Moreover, a PET study revealed microglial activation in hippocampus (80), which is positively related to neuronal integrity (109). Frustratingly, it remains difficult to conclude an absolute statement based on these limited studies.

Dysfunctional astrocytes and microglia reflect abnormal neuroinflammatory response in mood disorders. Below, we will discuss the astrocyte-microglia crosstalk and its pivotal role in affective immunology.

## The Astrocyte-Microglia Crosstalk in Neuroinflammation

### Overview of the Astrocyte-Microglia Crosstalk

Although both microglia and astrocytes belong to glia cells, they have very different origins; the former are CNS resident macrophages, while the latter are derived from neuroepithelial progenitors and serve as stromal cells (110, 111). To date, numerous cellular and molecular mechanisms of bidirectional communication between them have been shown (49, 112).

Astrocyte-derived IL-1 could activate microglia via permeabilizing the BBB. Besides, astrocytes can release inflammatory cytokines and chemokines, such as IL-15 (113), IL-33 (114), migration inhibitory factor (MIF) (115), and ATP (116), to directly enhance microglial abilities like migration, engulfing apoptotic cells, phagocytosing extracellular matrix, and pruning synapses. Similarly, microglia could influence astrocytic activity by releasing ATP (33), complement factor C1q, IL-1α, TNF (49), transforming growth factor-beta (TGF-β), vascular endothelial growth factor-β (VEGF-β) (50), and insulin-like growth factor 1 (IGF-1) (14). Moreover, astrocytes and microglia communicate by coordinated response using common soluble factors [including norepinephrine (117, 118), purines (119), and circulating bacterial metabolites from gut microbiome (47, 50)], consistently impacting the neuronal activity. Last but not least, there is limited evidence to explain the relatively stable proportions of astrocytes and microglia. Researchers have hypothesized that communication between astrocytes and microglia performs an essential role in balancing their proportionate numbers (120, 121).

Taken together, the microglia and astrocytes function synchronously and complementarily during various physiological and pathological processes (14, 122), including synaptic formation and remolding, BBB regulation, homeostasis, and immune response (123). Once the astrocyte-microglia crosstalk is perturbed, pathological events occur.

### The Astrocyte–Microglia Crosstalk During Innate Immune Response

**Figure 2:**
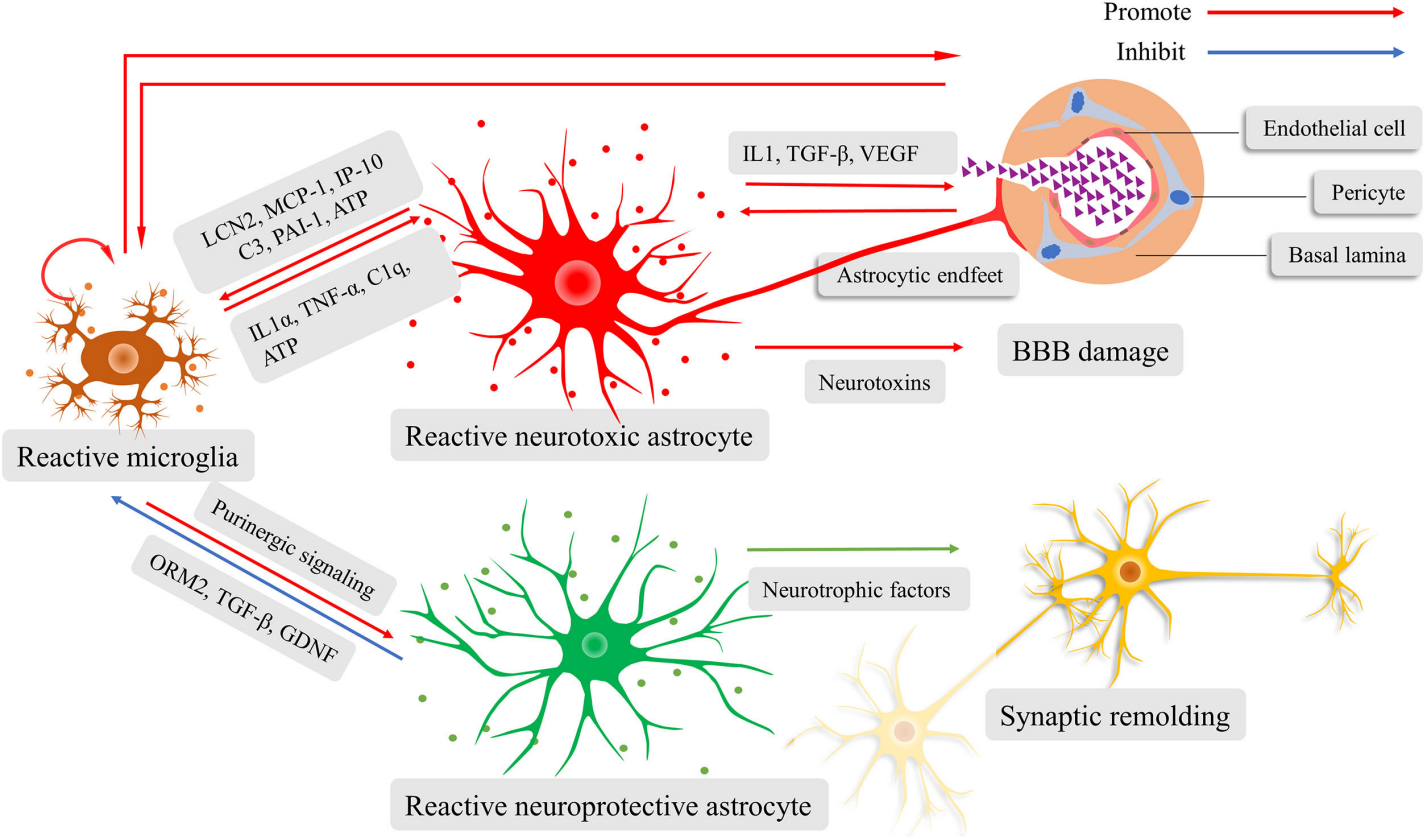
Schematic illustration of the fine interaction between astrocytes and microglia during neuroinflammation. Reactive microglia activate and determine the phenotypes of astrocytes, ranging from neurotoxic to neuroprotective. The reactive neurotoxic astrocytes promote the capacity of microglial activation, motility, and phagocytosis, while weakening the blood-brain barrier (BBB) and prune synapses. Increased BBB permeability facilitates the recruitment of immune cells and diffusion of inflammatory cytokines, amplifying neuroinflammatory response. The reactive neuroprotective astrocyte can lead to microglial inactivation, synaptogenesis, and scar formation.

Both astrocytes and microglia actively participate in neuroinflammation by regulating the innate immune system (124). When the microglia sense danger signals with their motile protrusions, they immediately release cytokines and chemokines that lead to reactive astrocytosis. Interestingly, the phenotypes of reactive astrocytes, whether they are neuroprotective or neurotoxic, are determined by microglia-derived pro-inflammatory cytokines according to diverse pathological conditions (49, 119, 125). The neuroprotective reactive astrocytes are induced via purinergic signaling (119). They can release neurotrophic factors and secret proteins, resulting in synaptogenesis and scar formation (119). The scar protects brain tissue from invading of excessive inflammation response. Conversely, the neurotoxic reactive astrocytes increase the expression of multiple genes that are related to tissue damage and induction of proinflammatory mediators (112, 126). Astrocyte-derived proinflammatory molecules can increase BBB permeability, which contributes to the recruitment of immune cells and increased migration and phagocytosis of microglia (30). This thereby amplifies the inflammatory response. Many researches have shown the pivotal nature of lipocalin-2 (LCN2) (127), as well as monocyte chemoattractant protein 1/C-C motif chemokine ligand 2 (MCP-1/CCL2), IFN-γ inducible protein 10/C-X-C motif chemokine ligand 10 (IP-10/CXCL10) (128), complement factor C3 (129), and plasminogen activator inhibitor type 1 (PAI-1) (130) in enhancing microglial activity. Contrarily, at the late stage of inflammation, the reactive astrocytes attenuate microglial activation by orosomucoid-2 (ORM2) (131), TGF-β (132), and glial cell line-derived neurotrophic factor (GDNF) (133), and inhibits the microglial phagocytosis by pentraxin 3 (PTX3) (134), thereby limiting the neuroinflammation. Above all, the astrocyte-microglia crosstalk is crucial for moderating innate immune response; otherwise, the neuroinflammatory response would get out of control (Figure 2).

### Blood-Brain Barrier, Gut Microbiota, and the Astrocyte-Microglia Crosstalk

As stated earlier, the alteration in BBB permeability is a key mechanism of regulating neuroinflammatory response. This allows or restricts the entry of immune cells and peripheral inflammatory mediators into the parenchyma of CNS at different stages. As one of the essential components of BBB, astrocytes dynamically regulate its permeability through inflammatory cytokines and connexins on endfeet (135–137). For instance, reactive astrocytes become hypertrophic with a reduced number of connexins to weaken BBB during neuroinflammatory conditions (90). Besides, resting microglia is beneficial for relatively intact BBB, while reactive microglia tend to migrate and induce BBB breakdown (138). Meanwhile, the gut microbiota is essential for the integrity of BBB (139). Once barriers are breached, blood-derived signals, including circulating microbial metabolites from the gut microbiota, can enter the brain and act back on the astrocyte-microglia crosstalk (47, 50).

Specifically, both astrocytes and microglia express the aryl hydrocarbon receptors (AHRs) (50) and can sense tryptophan metabolites (46) and SCFA, respectively (47). The AHRs signaling creates an anti-inflammatory state by balancing the gene expression of TGF-α and VEGF-β (50). Moreover, the normal development of microglia is strongly associated with a full repertoire of gut microbiota in early life (47, 140, 141). Reciprocally, the astrocyte-microglia crosstalk has been found to influence intestinal permeability and microbiome profile (142).

As for mood disorders, overactive astrocyte-microglia crosstalk can increase the permeability of BBB and intestine, which facilitate the diffusion of various inflammatory cytokines and microbial metabolites, and further activate the astrocyte-microglia crosstalk. In this context, the feedforward mechanism amplifies the neuroinflammatory response. Therefore, we postulate AAMC at the heart of affective immunology.

## Crosstalk Between Astrocytes and Microglia in the Affective Immunology: Does It Play Key Role?

Given the abundant evidence indicating the significant role of neuroinflammation in affective immunology, including inflammatory cytokines and gut microbiota (38, 42, 43), postmortem studies and molecular imaging researches have revealed that AAMC participates in the development of mood disorders (Figure 1) (56, 72, 78, 80). Despite recent progress, the underlying mechanisms remain elusive. Given that recent data has shown that AAMC serves as an essential mediator of both inflammatory cytokines and gut microbiota (114, 142, 143), we propose the hypothesis that astrocyte-microglia crosstalk triggers emotional alteration through regulating neuroinflammatory response, and thus contributes to mood disorders. In this section, we summarize data from present literature and discuss the crosstalk between astrocytes and microglia, aiming to support our hypothesis. However, the limitations of this hypothesis are also presented, and the suggestions for future research are offered.

As mentioned above, reactive astrocytes and microglia have been reported in mood disorders. Their presence is strongly suggesting of increased neuroinflammatory response (32, 81, 87). Reactive astrocytes become hypertrophic with reduced gap junction proteins, and they release cytokines and chemokines (such as IL-1, VEGF-A, TGF-β, and MIF), which drives BBB disruption and enhances microglial activation, migration, and phagocytosis (88, 90, 112, 135, 137). The reactive microglia make a significant impact on the astrocytic transformation (neuroprotective or neurotoxic phenotypes) and capacity mediating by purine signaling and inflammatory cytokines (40, 49, 119). Moreover, the increased BBB permeability facilitates the infiltration of peripheral immune cells, circulating cytokines and microbial metabolic (30). This can promote astrocytic and microglial activity, thereby amplifying the neuroinflammatory response (46, 50). Indeed, the studies regarding upregulated cortical inflammatory cytokine further support the neuroinflammatory cascade in mood disorders (2, 40). The excessive neuroinflammatory response in mood disorders reflects AAMC, which results in detrimental impacts on the downstream processes of the astrocyte-microglia crosstalk, such as neurotransmission and synaptic remolding (33, 123).

To our knowledge, astrocytes express glutamate receptors and regulate glutamate homeostasis through exocytosis (clearance of excess glutamate) and endocytosis (glutamate re-storage and transportation) (144). Dysfunctional astrocytes account for the imbalanced glutamatergic neurotransmission and hence excitotoxicity seen in mood disorders (65, 116). Arguably, microglia serve as a coordinator of astrocytes in regulating neurotransmission (33). Reactive microglia derive ATP and recruit astrocytes, resulting in an increase of glutamic release (33). In addition to ATP pathway, microglia-derived quinolinic acid known as an N-methyl-D-aspartate (NMDA) receptor agonist has been found to be upregulated, thereby contributing to the high-level glutamate in mood disorders (32). More importantly, inflammatory cytokines derived from AAMC (IL-1β, IL-6, and TNF-α) leads to downregulated expression and functionality of the excitatory amino-acid transporters 2 (EAAT2). This further attenuates the astrocytic ability of buffering and clearing the excessive glutamate (145). Conversely, the NMDA receptor antagonist ketamine can reverse the AAMC in mood disorders and alleviate excitotoxicity during the neuroinflammatory response, and hence exert rapid antidepressant effect (98, 99).

On the other hand, the anti-depressive effect of ketamine should ascribe to a reversal of another downstream process of AAMC-triggered neuroinflammation, the synaptic remolding in mood disorders (63, 123, 146). Inflammatory cytokines produced by astrocytes and microglia, especially IL-1β, TNF-α, and IFN-α, can detrimentally affect the synaptogenesis by regulating the expression of genes involved in synaptic plasticity (123). In addition, complement factors C1q and C3, as well as anti-inflammatory cytokine– TGF-β, are critical mediators of synaptic pruning and refinement (147). The neuroinflammation-driven synaptic remolding results in abnormal neurocircuits in mood disorders (14).

Taken together, these findings suggest the exciting possibility that AAMC can be a promising target for preventing and treating mood disorders. However, there are several limitations to the hypothesis. For example, the other components of BBB, such as endothelial cells and pericytes, are crucial for homeostatic brain and neuroinflammation (30). Besides, the fine interaction between neurons and the astrocyte-microglia crosstalk is vital for mental health (118, 148). Further discussion should take these factors into account to improve the hypothesis.

## Conclusion

This study highlights the vital role of the astrocyte-microglia crosstalk in affective immunology and posits that AAMC triggers emotional changes by modulating neuroinflammatory response. This thus contributes to the development of mood disorders. Most of the supporting evidence discussed here comes from human studies. Few animal experiments are cited as proof to elucidate the cellular and molecular mechanism, bearing in mind that animal models are insufficient to reflect the pathophysiology of mood disorders. However, postmortem studies might omit transient pathological alterations of astrocyte-microglia crosstalk seen in mood disorders, due to small sample size and confounding factors including age, disease duration, phenotype, medication use, postmortem interval, and duration of tissue storage. Fortunately, molecular imaging can detect the transient abnormalities of the astrocyte-microglia crosstalk *in vivo* and hopefully be applied to the individualized treatment of disorders.

## Data Availability Statement

The original contributions presented in the study are included in the article/supplementary material, further inquiries can be directed to the corresponding author/s.

## Author Contributions

LY, YZ, HJ, and ST wrote the paper and made the original figures. AS, LY, and YQ critically revised the texts and figures. All authors read and approved the final manuscript.

## Conflict of Interest

The authors declare that the research was conducted in the absence of any commercial or financial relationships that could be construed as a potential conflict of interest.

## Abbreviations

AAMC: altered astrocyte-microglia crosstalk
CNS: central nervous system
BBB: blood-brain barrier
MCP: monocyte chemoattractant protein
IL: interleukin
TGF: transforming growth factor
NK: nature killer
CRP: C reactive protein
HPA: hypothalamic-pituitary-adrenal
MDD: major depression disorder
BD: bipolar disorder
IFN: interferon
TNF: tumor necrosis factor
SCFA: short-chain fatty acids
MIF: migration inhibitory factor
VEGF: vascular endothelial growth factor
CCL: C-C motif chemokine ligand
GDNF: glial cell line-derived neurotrophic factor
PTX3: Pentraxin 3
AHR: aryl hydrocarbon receptor
PFC: prefrontal cortex
ACC: anterior cingulate cortex
TSPO: translocator protein
GFAP: Glial fibrillary acidic protein
TMS: transcranial magnetic stimulation
CSF: cerebrospinal fluid
BA: Brodmann area
ATP: adenosine triphosphate
IGF-1: insulin-like growth factor 1
LCN2: lipocalin-2
ORM2: orosomucoid-2
IP-10: IFN-γ inducible protein 10
CXCL10: C-X-C motif chemokine ligand 10
PAI-1: plasminogen activator inhibitor type 1
HLA-DR: Human leukocyte antigen D-related
AQP-4: aquaporin-4
CHI3L1: chitinase-3-like protein 1
NMDA: N-methyl-D-aspartate
EAAT2: excitatory amino-acid transporters 2.
